# Association between daily activities and fall in older adults: an analysis of longitudinal ageing study in India (2017–18)

**DOI:** 10.1186/s12877-022-02879-x

**Published:** 2022-03-14

**Authors:** Aarti Nagarkar, Snehal Kulkarni

**Affiliations:** Department of Health Sciences, Savitribai Phule Pune University, Pune, Maharashtra India

**Keywords:** Activities of daily living, Fall, Functionality, LASI, India

## Abstract

**Background:**

Declining functionality affects an individual’s musculoskeletal integrity increasing the risk of fall and disability. Individuals with severe functional limitations are 5 times more likely to experience a fall. Thus, this paper investigated the association between functional decline and falls in older adults.

**Methods:**

This study uses secondary data from the Longitudinal Aging Study in India (2017–18). A total of 31,477 people over the age of 60 are included in the study. Descriptive statistics and bivariate analysis were performed to determine the association between activities of daily living (ADL), instrumental activities of daily living (IADL) and fall. Adjusted odds ratio was used to determine the association of ADL and IADL with fall while controlling for age, gender, balance and gait impairments.

**Results:**

The study reported 6352 fall episodes in 3270 participants aged 60 and above, over a period of 2 years. More than 30% of participants reported difficulty in ADL and IADL. Age and gender adjusted odds of fall were higher in participants with difficulty in more than 4 ADLs (AOR:1.32; CI:1.08 – 1.67) and in more than 2 IADL (AOR: 1.39; CI:1.02 – 1.89). Similarly, the odds of fall were higher for difficulty in ADL (AOR:1.31; CI:1.11 – 1.73) and IADL (AOR of 1.18; CI:1.07 – 1.29) controlling for gait and balance impairment. Difficulty in pushing-pulling objects (AOR: 1.30; CI: 1.15 – 1.46 & AOR: 1.40; CI: 1.21–1.61) and getting up from the chair (AOR: 1.12; CI:1.01–1.26 & AOR: 1.27; CI: 0.99 – 1.26) was significantly associated with fall when adjusted for age, gender and balance and gait parameters.

**Conclusions:**

This study provides the new insights into the association of fall and risk of functional decline, especially difficulty in pushing and pulling objects and getting up from a chair, can be incorporated in the primary screening of fall risk assessment.

## Background

Functional decline is characterised by an increased inability to perform basic activities of daily living (ADL) and instrumental activities of daily living (IADL) [1]. Over 45% of older adults aged 60 and above report difficulty in performing everyday activities [2]. Decline in functioning affects a person's musculoskeletal integrity and body composition, lowering their ability and increasing their risk of falling, disability, and loss of independence [3, 4]. More than half of all falls occur while performing common daily activities such as walking, arising from chair, or transferring [5, 6]. Other factors such as advancing age, multimorbidity, vision and hearing impairment, gait and balance impairment are also reported to increase the risk of fall in older adults [7, 8].

Nearly, one-third of community-dwelling older adults fall, and 10–15% of them endure an injury, making falls a global health concern [9, 10]. Studies have identified activity limitations as one of the risk factors for falls as well as the consequences of falls [11, 12]. A fivefold increase in fall rates has been observed in those with severe functional limitations [4]. Despite this, the lack of studies from developing countries limits our understanding of the association between functionality and falls experienced by the older population in low-middle income countries. India is the second-most populous country in the world and will host 173 million people above 60 years of age by 2026 [13]. In recent years, several studies from India have investigated the prevalence and risk factors for falls. The prevalence of fall reported in these studies ranges between 14–53 per cent and the risk factors include socio-demographic factors, previous falls, visual impairment, reduced functionality, chronic illness or pain, cognitive impairment, environmental hazards, gait or balance impairment, and many more [14–16]. Without preventive interventional efforts in place, the burden of falls, injury, and disability will increase exponentially in the coming decades [17]. Thus, older adults must remain active, mobile, and care for themselves as it will reduce the cost of care. As a result, a better understanding of the relationship between functional level, activity limitation, and fall will aid in the development of relevant strategies and policies. Therefore, the present study aims to ascertain the association between functional decline and falls among older adults in India using data acquired from the Longitudinal Ageing Study in India.

## Methodology

### Data and sample

Data used in the current study is collated from the First Wave of the Longitudinal Ageing Study in India (LASI) conducted during 2017–18. A multistage stratified area probability cluster sampling design was used in LASI to select the observational units. A three and four-stage sampling design was adopted for rural and urban areas, respectively. A household with at least one respondent aged 45 and above was selected as a sample from all the states and UTs of India except Sikkim. The survey covered 72,262 individuals aged 45 and above and their spouses irrespective of age. LASI provides vital information on demography, and self-reported information on chronic health conditions, symptoms-based health conditions, functional health, mental health (cognition and depression), household economic status, healthcare utilization and insurance, family and social network. The present study uses the variables of activities of daily living and fall in past 2 years to investigate the association between them. From the 72,262 participants, the final sample included for the analysis is 31,477 individuals aged 60 years and above.

### Dependent variable

Fall: The dependent variable for the current study was an episode of fall. Participants aged 60 or over were asked whether they had fallen in the last two years for any reason (excluding road traffic accidents and occupational falls). The responses were coded as ‘1 = yes’ and ‘2 = no’.

### Independent variables

The main independent variable is the activities of daily living (ADL) and instrumental activities of daily living (IADL). The original questionnaire provides a list of 9 ADL activities and 7 IADL activities. From them 8 activities were included for ADL and 6 items were included to measure IADL. The selected activities; walking, sitting for 2 h or more, getting up from a chair, climbing, stooping/kneeling/crouching, reaching or extending arms above the shoulder, pushing or pulling large objects, and lifting weights were included for measuring ADL as they require postural stability and contribute to the risk of falling. Difficulty in IADL was assessed using 6 components; difficulty in shopping for groceries, making telephone calls, medications, managing money such as paying bills and keeping track of expenses, doing work around the house or garden, and getting around or finding an address in an unfamiliar place. The 7^th^ item ‘Preparing meals’ was not considered for analysis, as only a few participants (13%) reported difficulty in this activity. These ADL and IADL measures showed good internal consistency with Cronbach’s alpha of 0.872 for ADL 0.854 for IADL respectively, indicating high reliability of both measures.

The responses were coded as 0 = no difficulty and 1 = difficulty. ADL categories were computed by adding all the 8 ADL activities mentioned above and categorized as 0 = no difficulty in any activity, 1 = difficulty in up to 4 activities, and 2 = difficulty in more than 4 activities. IADL categories were computed similarly by adding the 6 IADL activities and categorized as 0 = no difficulty in any IADL, 1 = difficulty in 2 or less than 2 IADLs, and 2 – difficulty in more than 2 IADLs.

Further age, gender, hearing and vision impairment, balance, and gait parameters were included as independent variables in the analysis. Age was categorised as 1 = 60–69 years, 2 = 70–79 years, 3 = 80 and above. The presence of visual (*n* = 31,382) and hearing impairment (*n* = 31,380) was coded as 0 = no, 1 = yes. Balance impairment (*n* = 24,969) was coded as 0 = no, 1 = yes. Gait speed (meters/second) (*n* = 26,767) was coded 0 = 0.85 and above m/s, 1 = 0.60 – 0.84 m/s and 2 = gait speed between 0–0.59 m/s, highest score given to slowness. Different variables have different number of missing data therefore percentages are computed based on the “n” for these variables.

#### Ethics

Although this paper is based on secondary data analysis, the original study reports compliance with research ethics. Details are available in the report published on the website Longitudinal Ageing Study in India (LASI) Wave-1, India Report [18].

#### Statistical analysis

Descriptive statistics (frequencies, percentages, means, and standard deviations (SD) were used for all participant characteristics and associated factors of self-reported falls. With self-reported falls (categories: no versus yes) as the dependent variable, bivariate and multivariate binary logistic regression analyses were executed to examine the association with different independent variables. Independent variables included in the regression models were the number of difficulties reported in performing ADL and IADL, age, gender, visual and hearing impairment, balance impairment, and slow walking speed. Further to establish a more robust association between ADL difficulty, IADL difficulty, and fall, multivariate regression was used to control for the other potential confounding risk factors of fall. The first model was adjusted for age and gender, 2^nd^ model was adjusted for balance and gait speed. Variables were inputted into the model using the forced entry for detailed analyses. Results were considered statistically significant with 95% confidence intervals and a *p*-value of < 0.05 and *p*-value of  < 0.01. Initially, bivariate analyses were conducted and independent variables that were found statistically significant were included in multivariate analysis.

## Results

There were 15,106 (48%) males and 16,371 (52%) females among the 31,477 participants. The participants' mean age was 68.8 ± 7.51 years, and they ranged in age from 60 to 116 years. Sixty-three percent of the participants were married, 29.6% were currently working, and 24% had less than seven years of education. The vast majority of participants (66%) lived in rural areas and about 54% of the participants did not ever attend school.

Table 1 shows the proportion of falls and their distribution across the risk factors in the study population. Out of the 31,477 older adults, 3270 (10.38%) participants reported having experienced 6352 falls in the past 2 years. Out of the 3270 fallers, 1863 were women and 1407 were men. Of the 3270 participants who had fallen, 1729 had experienced a single fall while 1541 participants had experienced 2 or more fall episodes. Among the 3270 fallers, 1369 (41.90%) participants experienced injurious falls needing medical attention, and 354 (11.4%) fall episodes resulted in fractures. As seen from Table 1 women reported higher odds (OR: 1.30; CI:1.21 – 1.40) of fall as compared to men. The odds of fall increased in participants above 80 years of age (OR: 1.15; 1.02–1.30) as compared to those below 80 years. More than 50% had slow gait speed (less than 0.8 m/s) and 22% were not able to complete the balance test. The odds of falls among participants with slow gait speed was 1.31(1.17 – 1.46) higher than their counterparts. Participants with balance impairment reported greater odds (OR: 1.28; CI: 1.18 – 1.37) as compared to those with no balance impairment. Higher odds of fall were reported by participants with hearing impairment (OR: 1.25; CI: 1.09 – 1.56) and vision impairment (OR: 1.11; CI: 0.98 – 1.31) as compared to participants with no impairment.

**Table 1:** Population characteristics and proportion of fall across all the variables

Variable	Categories	Frequency n (%)	Proportion of fall	Odd ratio (95%CI)
No. of respondents reporting Fall in past 2 years	Yes	3270 (10.4)	-	-
	No	25,019 (88.4)		
Gender	Male	15,106 (48)	1407(9.31)	0
	Female	16,371(52)	1863(11.37)	1.30 (1.21 – 1.40) *
Age	60–69	18,979(60.3)	1934(10.19)	0
	70–79	9108(28.9)	961(10.55)	1.05 (0.96– 1.14) *
	80 +	3390(10)	375(11.06)	1.15 (1.026– 1.30) *
Place of residence	Urban	10,747 (34.1)	933 (8.7)	0
	Rural	20,730 (65.9)	2337 (11.3)	1.37 (1.26 – 1.48) *
Ever attended school	Yes	14,583 (46.3)	1436 (10.9)	0
	No	16,894 (53.7)	1834 (12.1)	1.13 (1.05 – 1.21) *
Visual impairment	No	13,995(44.6)	1344(9.6)	0
	Yes	17,387(55.4)	1926(11.07)	1.11 (0.98 – 1.31) *
Hearing impairment	No	28,225 (89.9)	2871(10.17)	0
	Yes	3155 (10.1)	399(12.6)	1.25 (1.09 – 1.56) *
Balance impairment	No	19,488(78.0)	1380(7.08)	0
	Yes	5481(22.0)	1890(34.48)	1.28 (1.18 – 1.37) *
Gait speed	above 0.84	6592(24.6)	634(9.61)	0
	0.60–0.84	13,420(50.1)	1363(10.15)	1.07 (0.97 – 1.19) *
	0–0.59	6755(25.2)	1273(18.84)	1.31 (1.17 – 1.46) *

^*^p-value < 0.05. The reference categories were male, age group 60–69 years, no visual and hearing impairment, no balance impairment and gait speed above 0.8 m/s. Note that the percentage are computed based on the total number of observations for each variable

Table 2 shows the crude odds ratio of each ADL and IADL items for fall. The results show that difficulty in all the activities of ADL and IADL had significantly higher odds for fall at *p*-value of less than 0.05. The highest odds of fall were reported in those with difficulty in pushing-pulling objects (OR:1.79; CI: 1.66 – 1.93), climbing stairs (OR:1.75; CI: 1.62 – 1.89), arising from a chair (OR:1.65; CI: 1.51 – 1.75) and stooping (OR:1.63; CI: 1.53 – 1.78), difficulty with grocery shopping (OR: 1.46; CI: 1.34 – 1.59) and management of medications (OR: 1.45; CI: 1.31 – 1.60). Higher odds were observed in those with difficulty in more than 4 tasks (OR 2.32; CI: 2.09 – 2.56) and in participants with difficulty in more than 2 IADLs (OR:1.72; CI: 1.49 – 1.97) as compared to those reporting no difficulty.

**Table 2:** Association (odds ratio) between falls and ADLs / IADLs

Variables	Categories	N	Proportion of fall	Crude OR (95% CI)
Pushing-Pulling objects	No difficulty	15,420 (49.0)	1231 (7.98)	0
	Difficulty	16,057 (51.0)	2039 (12.69)	1.79 (1.66 – 1.93) *
Climbing	No difficulty	14,266 (45.3)	1136 (7.96)	0
	Difficulty	17,211 (54.7)	2134 (12.39)	1.75 (1.62 – 1.89) *
Arising from chair	No difficulty	18,193 (57.8)	1590 (8.73)	0
	Difficulty	13,284 (42.2)	1680 (12.64)	1.65 (1.51 – 1.75) *
Stooping	No difficulty	13,848 (44.0)	1133 (8.18)	0
	Difficulty	17,629 (56.0)	2137 (12.12)	1.63 (1.53 – 1.78) *
Walking	No difficulty	21,178 (67.3)	1974 (9.32)	0
	Difficulty	10,299 (32.7)	1296 (12.58)	1.51 (1.40 – 1.62) *
Sitting	No difficulty	19,390 (61.6)	1775 (9.15)	0
	Difficulty	12,087 (38.4)	1495 (12.36)	1.50 (1.32 – 1.67) *
Carry weights	No difficulty	19,662 (62.5)	1805 (9.18)	0
	Difficulty	11,815 (37.5)	1465 (12.39)	1.50 (1.39 – 1.62) *
Reaching objects	No difficulty	25,142 (79.9)	2461 (9.78)	0
	Difficulty	6335 (20.1)	809 (12.77)	1.47 (1.35 – 1.60) *
Shopping grocery	No difficulty	24,791 (78.8)	2418 (9.7)	0
	Difficulty	6686 (21.2)	852 (12.74)	1.46 (1.34 – 1.59) *
Household garden	No difficulty	24,113 (76.6)	2351 (9.74)	0
	Difficulty	7364 (23.4)	919 (12.47)	1.43 (1.32 – 1.55) *
Medications	No difficulty	27,159 (86.3)	2713 (9.98)	0
	Difficulty	4318 (13.7)	557 (12.89)	1.45 (1.31 – 1.60) *
Finance	No difficulty	24,067 (76.5)	2360 (9.80)	0
	Difficulty	7410 (23.5)	910 (12.28)	1.38 (1.27 – 1.50) *
Telephone calls	No difficulty	23,369 (74.2)	2269 (9.7)	0
	Difficulty	8108 (25.8)	1001 (12.34)	1.38 (1.27 – 1.49) *
ADL Difficulty	None	8407 (26.7)	568 (6.75)	0
	Less than 4	11,496 (36.5)	1182 (10.28)	1.62 (1.46 – 1.80) *
	More than 4	11,574 (36.8)	1520 (13.13)	2.32 (2.09 – 2.56)
IADL difficulty	None	18,573 (59.0)	1666 (8.97)	0
	Less than 2	10,785 (34.3)	1331 (12.34)	1.49 (1.38 – 1.61) *
	More than 2	2119 (6.7)	273 (12.88)	1.72 (1.49 – 1.97) *

^*^p-value < 0.05; reference categories for all the variables were ‘no difficulty’

Table 3 describes the association between ADL, IADL difficulty, and fall using multivariate regression. Model 1 shows results after adjusting for biological factors (age and gender) and Model 2 shows the odd ratio after adjusting for physical factors (balance and gait parameters). In both the models, difficulty in pushing and pulling objects (AOR:1.30; CI: 1.15 – 1.46) in model 1; AOR: 1.40; CI: 1.21 – 1.61) was found be to significantly (*p* < 0.01) associated with fall. Similarly, difficulty in arising from chair (AOR: 1.12; CI: 1.01 – 1.26) and AOR: 1.27; CI: 0.99 – 1.26) was significantly associated (*p* < 0.05) with fall after controlling for age, gender, balance and gait impairment. The odds of having difficulty in climbing stairs (AOR:1.13; CI:1.01- 1.27) was significantly associated with fall in model 1 but no significant association was observed in model 2. Those with ADL difficulty (up to 4 or more than 4) and IADL difficulty (up to 2 and more than 2) had significantly higher odds of falling after adjusting for age, gender, and physical factors. Significant association of individual items on IADL scale in univariate analysis did not sustain the statistical association in multivariate analysis.

**Table 3:** Association (adjusted odds ratio) of ADLs and IADLs with falls controlling for confounding factors

Variables	Categories	Adjusted for age and gender (model 1)	Adjusted for Balance and gait (model 2)
		OR 95% CI	OR 95% CI
Pushing-Pulling objects	No difficulty	0	
	Difficulty	1.30 (1.15 – 1.46) **	1.40 (1.21 – 1.61) **
Climbing flight of stairs	No difficulty	0	
	Difficulty	1.13 (1.01– 1.27) *	–
Arising from chair	No difficulty	0	
	Difficulty	1.12 (1.01 – 1.26) *	1.27 (0.99 – 1.26) *
ADL difficulty	None		
	Up to 4	1.12 (1.04 –1.43) *	1.21 (1.10 – 1.49) *
	More than 4	1.32 (1.08 – 1.67) *	1.31 (1.11 – 1.73) *
IADL difficulty	None		
	Upto 2	1.15 (1.06 – 1.26) **	1.16 (1.06 – 1.28) *
	More than 2	1.39 (1.02 – 1.89) *	1.18 (1.07 – 1.29) **

Model 1: Adjusted by age and gender; Model 2: adjusted for balance impairment and slow gait speed

^*^ Significance at *p* < 0.05,

^**^ Significance at *p*-value < 0.01

## Discussion

This study documented 6352 fall episodes in 3270 participants aged 60 and above, over a two-year period. The results show that increasing age, female gender, slow gait speed and impaired balance are associated with fall. Gait and balance disorders are among the most common causes of fall in older adults [19, 20]. With age, natural loss of muscle mass may lead to loss of balance and coordination thus, affecting gait and increasing the risk of recurrent falls in older adults [21]. More than 30% of the participants reported difficulty in performing ADLs and IADLs. Higher odds of fall were reported in participants with difficulty in more than 4 ADLs and 2 IADLs as compared to those reporting no difficulty. These findings are concurrent to the findings of other studies and imply that ADLs and IADLs have significant associations with falls in older adults [22, 23]. As age and gender, gait and balance impairment affect fall, we independently adjusted the results for these factors and only two ADLs consistently demonstrated a significant association with fall incidence.

An ADL which was significantly associated with fall was difficulty pushing and pulling. The mechanism of posture during pushing and pulling requires the application of significant force and the preservation of the centre of gravity (COG). Thus, when a person pushes or pulls an object, even a minor disturbance can cause a shift in COG, resulting in loss of balance, stumbling, and eventual fall [24, 25]. Because of this, we believe older adults should be particularly careful or, if possible, refrain from pushing-pulling things, as this activity puts them at an increased risk of falling. The second significant association was with difficulty in arising from a chair, also known as sit-to-stand (STS) action, which is a transition movement in which the centre of mass moves from a stable to a less stable position with overextended lower extremities. The ability to execute an STS movement is an important skill. The inability to perform this basic skill in older adults can result in impaired mobility, falls, or institutionalisation [26]. Previous studies have shown that difficulty in arising from a chair is associated with reduced lower-extremity muscle strength, poor balance, slower reaction time, and increased risk of falling [27, 28]. Drummond A et al. [29] in their study reports that difficulty in rising from a seated position may increase the risk of all falls by 41% among older adults in nursing homes. As a result, arising from a chair or sit-to-stand action can be recognised as an important measure in assessing fall risk. Perhaps this finding validates the use of the arising from chair measure as a screening measure.

### Strengths and limitations of the study

This paper has strengths as well as some limitations. The study's main strength was the large sample size and national-level representation of the Indian older adults. One of the strengths is probably the first time fall and certain attributes related to fall are recorded and analysed for Indian elderly. A limitation is that the results are cross-sectional and some variable shaving missing data. However, the authors believe that the findings are nevertheless of value. Our study only focused on the association between the functional limitation and reporting of fall. However, evidence suggests that a potential bi-directional association may exist between functional decline and fall (11,12). Despite this limitation, we feel that these results have a programmatic value. Furthermore, functionality data was just a set of dichotomous responses (yes or no) which limits the interpretation of data. For understanding finer differences in daily activities, future research may include ordinal response scale.

Our findings emphasise that functional decline is an important aspect of falls in elderly. There were a significant number of participants reporting difficulty performing daily activities and having an increased risk of falling. The results may be useful for community screening and identifying older adults who may be at risk of falls. Falls in older adults lead to a chain of catastrophic events that may result in dependency, disability, or death. In low- and middle-income countries, where resources are scarce, fall prevention programs may seem impossible to implement. It is much better to use the pointers that anticipates a future fall than relying only on chance. Lastly, findings of the study suggest that any fall prevention programme need to be tied to the fundamental goal to improve functionality and well-being of elderly in low-middle income countries.

## Data Availability

The study uses a secondary data which is available on reasonable request through https://www.iipsindia.ac.in/content/lasi-wave-i.

## Abbreviations

ADL: Activities of daily living
AOR: Adjusted odds ratio
CI: Confidence interval
IADL: Instrumental activities of daily living
LASI: Longitudinal Aging Study in India
OR: Odds ratio
